# Cisplatin Eligibility Issues and Alternative Regimens in Locoregionally Advanced Head and Neck Cancer: Recommendations for Clinical Practice

**DOI:** 10.3389/fonc.2019.00464

**Published:** 2019-06-11

**Authors:** Petr Szturz, Valerie Cristina, Ruth Gabriela Herrera Gómez, Jean Bourhis, Christian Simon, Jan B. Vermorken

**Affiliations:** ^1^Medical Oncology, Department of Oncology, Lausanne University Hospital (CHUV), Lausanne, Switzerland; ^2^Radiation Oncology, Department of Oncology, Lausanne University Hospital (CHUV), Lausanne, Switzerland; ^3^Department of Otolaryngology - Head and Neck Surgery, Lausanne University Hospital (CHUV), Lausanne, Switzerland; ^4^Faculty of Medicine and Health Sciences, University of Antwerp, Antwerp, Belgium; ^5^Department of Medical Oncology, Antwerp University Hospital, Edegem, Belgium

**Keywords:** head and neck cancer, chemoradiotherapy, cisplatin, cetuximab, targeted therapy, immunotherapy, clinical trials, practice recommendations

## Abstract

Well-designed randomized trials provide the highest level of scientific evidence to guide clinical decision making. In chemoradiotherapy of locally advanced squamous cell carcinoma of the head and neck (SCCHN), data support the use of three cycles of 100 mg/m^2^ cisplatin given every 3 weeks concurrently with conventionally fractionated external beam radiotherapy, although a full compliance with all three cycles is reserved to only about two thirds of initially eligible cases. On an individual patient level, practicing oncologists have to determine whether the patient is a suitable candidate for this treatment or whether contraindications exist. In the latter case, an adequate alternative has to be offered. In this regard, to facilitate triaging of medical information, we reviewed available publications on this topic and prepared practice-oriented recommendations for systemic treatment concurrent to definitive and post-operative radiotherapy. Even if no contraindications for the standard-of-care cisplatin apply, clinicians may opt for alternative regimens by adjusting the peak dose, cumulative dose, or timing of cisplatin. Relative contraindications pose the major issue in clinical practice, as very limited data is available in the literature and final decisions are usually based on an expert opinion or retrospective cohort studies. In the case of absolute interdiction of cisplatin, several alternative regimens incorporating carboplatin, 5-fluorouracil, cetuximab, and docetaxel are available. At the same time, it should be kept in mind that radiotherapy alone represents a viable option with hyperfractionation being particularly beneficial in the definitive management of limited nodal disease. Ideally, all treatment propositions should be discussed within multidisciplinary tumor boards taking into account the patient- and disease-related characteristics as well as local logistics and reimbursement policies.

## Introduction

Locoregionally advanced disease is still the most frequent clinical manifestation in patients with squamous cell carcinoma of the head and neck (SCCHN). In this setting, chemoradiotherapy offers an effective non-surgical approach as primary treatment, or alternatively, it can be delivered with adjuvant intent after a curative resection (1). Whether being part of bimodality or trimodality management, chemoradiotherapy usually comes at the cost of substantial acute and late toxicity, and it has been subject of numerous clinical trials to establish a treatment schedule with a reasonable compromise between its tumoricidal activity on the one hand and dose-limiting side effects on the other (2). This paper sets out to present the current standard-of-care chemoradiotherapy regimen in non-nasopharyngeal mucosal head and neck cancer along with other commonly used protocols for which a lower level of clinical evidence applies. Based on this theoretical framework, practice-oriented recommendations were conceptualized focusing primarily on systemic treatment. The different treatment options were categorized by clinical settings (definitive or post-operative) and by the presence or absence of contraindications to the standard-of-care treatment (absolute or relative). In addition, to rate the quality of evidence and strength of recommendations of each schedule mentioned here, the European Society for Medical Oncology (ESMO) grading consensus system was adopted (Table 1) (3). However, precise clinical, radiological, and pathological criteria used to select cases suitable for definitive or adjuvant chemoradiotherapy are not covered in this article. Furthermore, enrolment of patients in clinical research is highly recommended whenever a well-designed randomized trial opens for recruitment.

**Table 1:** Grading of the level of clinical evidence and strength of recommendation for clinical practice according to the ESMO consensus guidelines (3).

**Level of evidence**	
I	≥1 large well-conducted randomized control trial or meta-analyses of such trials
II	Randomized control trials with a suspicion of bias or meta-analyses of such trials
III	Prospective cohort studies
IV	Retrospective cohort studies or case-control studies
V	Studies without control group, case reports, and experts opinions
**Strength of recommendation**	
A	Strongly recommended
B	Generally recommended
C	Optional
D	Generally not recommended
E	Never recommended

## Defining the Standard of Care

The findings from four large randomized phase III trials established cisplatin-based chemoradiotherapy as the reference treatment both in the definitive and adjuvant treatment settings (3–8). The regimen consists of three infusions of 100 mg/m^2^ cisplatin given every 3 weeks concurrently with conventionally fractionated external beam radiotherapy. It represents a cost-effective, broadly available, and accessible treatment option (9, 10). The growing interest in de-intensification strategies investigated primarily in human papillomavirus (HPV)-positive oropharyngeal cancer has recently been dampened by the results of two phase III trials confirming the primacy of high-dose cisplatin against cetuximab (11, 12). Mounting evidence suggests that HPV-associated oropharyngeal cancer in men should be regarded as a separate entity with different biology and clearly a better prognosis (13). In economically developed countries, the prevalence of HPV-associated oropharyngeal cancer in men has been sharply increasing over the past three decades (14). At the same time, these regions have been the major force of clinical trial recruitment, enhancing their influence in academic communities (15). Thus, a notion may inadvertently be acquired that the changing epidemiologic landscape is uniform worldwide. However, the majority of patients with head and neck cancer still present with HPV-negative disease in which outcomes have been unsatisfactory calling for preservation of a sufficient treatment intensity. At present, HPV status has no predictive value in locoregionally advanced head and neck cancer.

Enrolling altogether 842 patients during the 1990s, two of the aforementioned trials were conducted in the definitive setting (4, 5). In a Head and Neck Intergroup trial, Adelstein et al. tested the benefit of chemotherapy as an adjunct to concurrent radiotherapy in patients with (mainly) unresectable squamous cell carcinoma of the oral cavity, oropharynx, hypopharynx, and larynx. The Radiation Therapy Oncology Group (RTOG) 91-11 trial, coordinated by Forastiere et al. was designed to compare the rates of larynx preservation between two chemoradiotherapy regimens (with induction or concurrent chemotherapy) and radiotherapy alone. Adelstein et al. (4) demonstrated a clear improvement of overall survival, the primary endpoint of the study (median: from 12.6 to 19.1 months, 5 year rates: from 14 to 26%). In the RTOG 91-11 trial, concurrent chemoradiation with 3 weekly cisplatin emerged as the optimal approach for larynx preservation, locoregional and distant controls, and disease-free survival. However, these benefits did not translate into overall survival advantage with 5 year rates being almost identical across all three treatment arms (about 55%). What is more, results of an updated publication after a median follow-up of 10.8 years caused a stir in the oncology community, suggesting a worse outcome in the concomitant chemoradiation treatment arm compared with the sequential treatment arm (*p* = 0.08) (16). Being attributed to an increase of deaths from non-cancer related causes probably due to unrecognized late toxicity, the correct interpretation is still a matter of debate. In this respect, it should be mentioned that RTOG 91-11 included only patients with glottic and supraglottic larynx cancer, in contrast to about 10% of such cases in the Intergroup study population. Therefore, subsite-specific impact on the results cannot be excluded.

In the post-operative setting, the RTOG 9501 and the European Organization for Research and Treatment of Cancer (EORTC) 22931 trials enrolled 793 patients with high-risk features in the pathology specimens between 1994 and 2000 (6, 7). The primary objectives were locoregional control and progression-free survival, respectively. In both trials, the addition of cisplatin to radiotherapy was associated with a significant enhancement of 5 year locoregional control and disease- or progression-free survival, but the prolongation of overall survival reached statistical significance only in EORTC 22931, being 53% vs. 40% (hazard ration [HR] 0.70, 95% confidence interval [CI]: 0.52–0.95, p = 0.02) at 5 years. In this context, special attention should be paid to patient selection criteria. An exploratory pooled analysis implied that a significant advantage of combined modality treatment was limited to patients with extracapsular spread and/or positive surgical margins. Importantly, the EORTC inclusion criteria defined microscopically involved margins as the presence of tumor at 5 mm or less, while RTOG 9501 did not allow such tolerance. Hence, it could be speculated to what extent this difference influenced the outcomes, above all its impact on overall survival. In any case, patients with close margins should be considered for adjuvant chemoradiotherapy. Of note, systemic treatment had no meaningful impact on distant control in these two trials, with rates varying between 80 and 75% irrespective of treatment cohort in the adjuvant, but also definitive settings.

Of further evidence has been the individual patient-based meta-analysis of 87 randomized trials, performed between 1965 and 2000 (17). This meta-analysis demonstrated that adding chemotherapy to locoregional treatment in locally advanced SCCHN was associated with an absolute survival advantage of 4.5% at 5 years (*p* < 0.0001). The conclusions on this benefit did not differ significantly between post-operative radiotherapy and definitive curative radiotherapy and with using either conventional or altered fractionation. However, chemotherapy protocols varied largely in this meta-analysis in that different drugs and different dose levels were applied. No preference for poly-chemotherapy including platin or 5-fluorouracil over mono-chemotherapy with cisplatin or vice-versa was noted. Single agent cisplatin appeared, therefore, to be one of the standard treatments in combination with radiotherapy. Most of the randomized trials in the analysis used a dose of cisplatin of 100 mg/m^2^ three times throughout the course of radiotherapy (cumulative dose of 300 mg/m^2^), and this came forward as the preferred and recommended option.

Two further variables remain to be addressed, i.e., toxicity and compliance. Adding cisplatin to radiotherapy was found to be associated with an increase in acute adverse events, both in terms of toxicity related primarily to the systemic treatment (gastrointestinal, hematological, neurological, and renal side effects) and toxicity owing mainly to radiotherapy (mucositis, dysphagia, and skin adverse events). Data on ototoxicity were not available. As an example, with the addition of high-dose cisplatin, the rate of severe acute mucositis almost doubled in EORTC 22931 (from 21 to 41%) and more than one third of patients developed severe acute dysphagia in RTOG 91-11. Unfortunately, in general, late toxicity reporting often suffers from inaccuracy and inconsistency (2). With that in mind, the cumulative incidence of late toxicity ranged between 20 and 40%, without a statistical correlation with the systemic treatment (6, 7, 16, 18). It was not surprising that the high rate of acute side effects came at the cost of decreased compliance. In fact, the proportion of patients who could receive all planned cycles of chemotherapy was between 61 and 85%.

## Decision-Making Process

The cisplatin-based concurrent chemoradiation protocol presented above is generally accepted as the reference for the definitive non-surgical and post-operative approaches in selected patients with locoregionally advanced SCCHN. At the same time, the efficacy is far from being satisfactory and toxicity is one of the major drawbacks. Nevertheless, the four randomized trials established level I evidence for its use supported by the individual patient-based meta-analysis, and no other regimen has proven to outperform this. The decision-making process gets complicated in the presence of patient-related characteristics hindering the employment of cisplatin. In their 2016 seminal work, Ahn et al. (largely opinion leaders from the Asia-Pacific region) summarized criteria for absolute contraindications and high-risk cases (19). Subsequently, these criteria were adopted for the purpose of the present work as absolute and relative contraindications. The original Ahns' criteria did not differentiate between palliative and curative settings. Herein, we focus on locally advanced disease where the addition of 3 weekly high-dose cisplatin to radiotherapy may save further patients' lives, and the absolute overall survival benefit at 5 years may be even higher than 10% (17). In this respect, the following modifications were made (Table 2).

**Table 2:** Absolute and relative contraindications to cisplatin in definitive or post-operative treatment of locally advanced head and neck cancer, modified from Ahn et al. (19).

**Clinical condition**	**Relative contraindications**	**Absolute contraindications**
Performance status	ECOG score = 2	ECOG score ≥ 3
Biological age	According to geriatric assessment and screening tools	ND
Renal dysfunction	Creatinine clearance 50–60 ml/min	Creatinine clearance <50 ml/min
Hearing impairment	Hearing loss or tinnitus grade = 1 or 2^a^, ^b^	Hearing loss or tinnitus grade = ≥ 3^a^
Neuropathy	Grade = 1^a^	Grade = ≥ 2^a^
Marrow, hepatic, respiratory, and cardiovascular, dysfunctions	Grade 2^a^ or Child-Pugh score = B^c^	Grade ≥ 3^a^ or Child-Pugh score = C^c^
Other comorbidities	Insulin-dependent diabetes mellitus, recurrent (pulmonary) infections, severe psychiatric disorders interfering with treatment compliance	Life-threatening conditions such as uncontrolled systemic infection or autoimmune disease
HIV/AIDS	CD4 count 200–350/μl^d^	CD4 count < 200/μl^d^
Nutritional status	Involuntary weight loss ≥ 20%	ND
Pregnancy and lactation	ND	First trimester of pregnancy^e^, lactation not recommended
Hypersensitivity to platinum agents	ND	Allergy to agents that contain platinum^f^ or mannitol
Previous platinum therapy	>200 mg/m^2^ or >3 cycles of TPF induction	ND
Drug interactions	Concomitant use of nephrotoxic drugs	ND
Socioeconomic status	Impaired social and economic support	ND

a *Based on the National Cancer Institute Common Toxicity Criteria version 4.0*.

b *Repeated audiometry exams may be indicated during the treatment*.

c *For hepatic impairment*.

d *World Health Organization definition*.

e *Fetal exposure to radiation, irrespective of the duration of pregnancy, increases the risk on developing malignancies in childhood and in addition is associated with abortion and intra-uterine death. Therefore, radiotherapy is preferably postponed until after delivery*.

f *If a skin test does not rule out cross-reactions among platinum agents*.

*HIV/AIDS, human immunodeficiency virus infection/acquired immune deficiency syndrome; ECOG, Eastern Oncology Cooperative Group; TPF, docetaxel, cisplatin, 5-fluorouracil; ND, not defined*.

First, the age limit of 70 years (calendar age) was removed because fit elderly individuals receiving full-dose treatment were shown to derive the same magnitude of clinical benefit as their younger counterparts (20). Thus, where applicable, our decisions should implement geriatric screening tools and if necessary complex geriatric assessment (21). Frailty as a surrogate marker for biological age represents a crucial factor in decision making related to older cancer patients. About 10% of the general senior population are expected to be frail. However, in the context of an oncologic disease, this proportion rises to over one half, comprising also vulnerable individuals, with not more than one third being fit. According to recently published clinical recommendations for systemic therapy of head and neck cancer in the elderly, fit patients should primarily be considered for high-dose 3 weekly cisplatin with curative intent, while treatment in those who are frail will rather consist of palliative measures such as palliative irradiation and/or palliative surgical interventions (e.g., tracheostomy, gastrostomy). In the intermediate group characterized by vulnerability, management follows the recommendations pertinent to the intermediate group with relative contraindications to high-dose cisplatin as explained further in this paper (22).

Next, pre-existing hearing impairment grade II was moved from absolute to relative contraindications. This condition belongs to the class-specific adverse events of cisplatin and can indeed be accelerated by such treatment. However, according to two large meta-analyses of 59 prospective trials, severe ototoxicity has not been common even with high cumulative doses of cisplatin, and the risk-benefit ratio on an individual patient basis can ultimately favor the standard, high-dose treatment (23, 24). Still, periodic audiometry exams might be indicated throughout the treatment course leading eventually to cisplatin interruption in some cases. Further modifications relative to the Ahn's criteria concerning organ dysfunctions, other comorbid conditions, and pregnancy are listed in Table 2.

**Figure 1:**
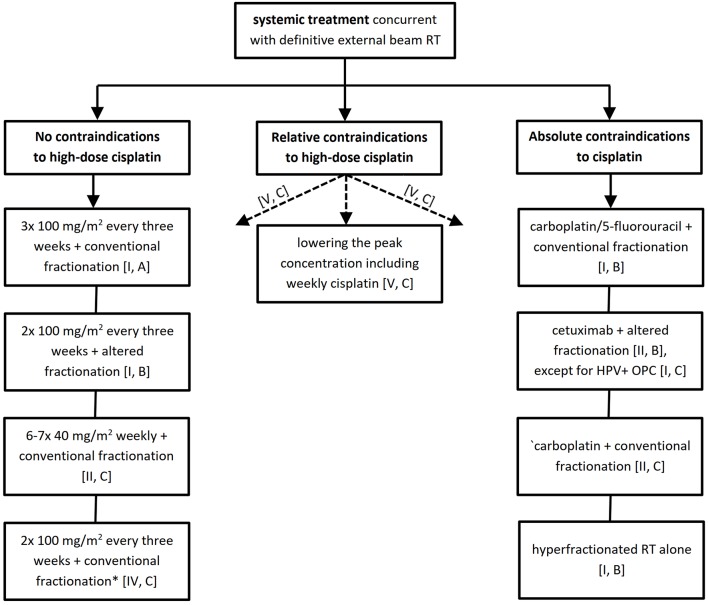
Systemic treatment recommendations for definitive chemoradiotherapy. *Particularly in human papillomavirus positive low risk or intermediate risk oropharyngeal cancer. RT, radiotherapy; HPV+ OPC, human papillomavirus positive oropharyngeal cancer.

**Figure 2:**
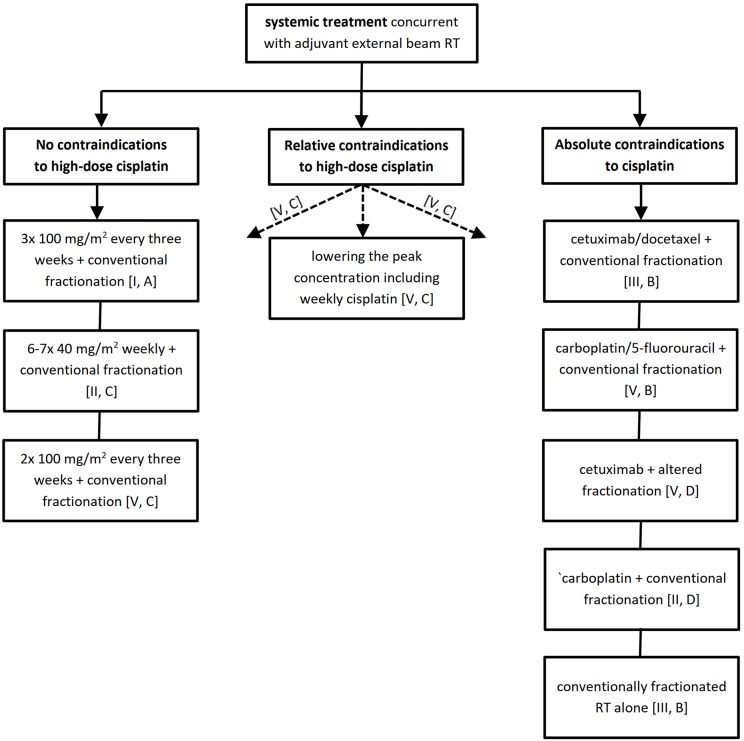
Systemic treatment recommendations for adjuvant (post-operative) chemoradiotherapy. RT, radiotherapy.

The bottom line is that patient and disease characteristics are crucial in decision making which should preferably be consensual within the frame of a multidisciplinary tumor board. To facilitate this task, we have elaborated a decision tree algorithm separately for the definitive and post-operative treatment settings available in Figures 1, 2 together with an overview of studies supporting the resulting level of evidence and grade of recommendation provided in the Tables S1, S2.

## Definitive treatment setting

### No Contraindications to High-Dose Cisplatin

The standard of care should be pursued whenever patients are in good general condition with few and/or mild comorbidities and are willing to adhere to the treatment program [I, A]. Alternatively, two cycles of 100 mg/m^2^ of cisplatin given in a 3–4 week interval concomitantly with altered fractionation radiotherapy may be considered [I, B] (12, 24, 25). On the other hand, current evidence is insufficient to prioritize weekly low-dose cisplatin protocols (26). Up to now, three prospective trials comparing survival outcome with weekly low-dose cisplatin-based chemoradiotherapy vs. radiotherapy alone have been published. The first two studies, enrolling a total of 275 patients, were conducted in the 1980s. Quon et al. chose a relatively low cumulative dose of cisplatin (7 ×20 mg/m^2^) being very probably responsible for the disappointing results. Median overall survival was even numerically worse in the combined modality arm (11.8 months vs. 13.3 months) (27). Sharma et al. doubled the target cumulative dose (7 ×40 mg/m^2^) leading apparently to better outcomes with a significant separation of overall survival curves (median: 27 months vs. no reached, *p* = 0.02). Nevertheless, the median follow-up period did not exceed 2 years and no information on late toxicity was provided (28). The third prospective study, a three-arm trial comparing two radiotherapy fractionation schedules with chemoradiation using up to 8 cycles of 30 mg/m^2^ cisplatin, was underpowered and had to be terminated prematurely due to poor accrual (199 out of 750 patients planned). The small improvement in locoregional control (*p* = 0.049) did not translate into significant overall survival improvement and the difference was only numerical (5 year rates: 56% vs. 36%) (29). Other prospective and retrospective trials exploring the weekly schedule are available but the data have been conflicting [II, C] (23). For further information regarding a comparison between the weekly and 3 weekly regimen please see below in a separate chapter.

Finally, retrospective observations in patients intended to receive three cycles of high-dose cisplatin suggest that a cumulative dose of 200 mg/m^2^ produces an adequate anti-tumor effect in terms of overall survival, especially in the prognostically favorable low-risk group of HPV positive oropharyngeal cancer, with higher doses possibly further improving locoregional control (11, 25, 30–32). At present, it is unclear whether dose escalation up to 300 mg/m^2^ brings additional survival advantage or whether this is offset by excessive toxicity responsible for an increase in non-cancer related deaths. Similarly, it remains unknown whether the progressively extending survival associated with 10 mg/m^2^ cisplatin increments in a range between 140 and 270 mg/m^2^, as demonstrated in a model based on 6 phase III trials, is due to the higher dose itself or to healthier patients better tolerating additional cisplatin delivery (33). Thus, even if two doses of 100 mg/m^2^ cisplatin given concurrently with conventional fractionation may be considered by some experts sufficient in the context of drug exposition [IV, C], clinicians should always ensure maximal comfort and supportive care for their patients and if toxicity permits, administration of the third cycle is indicated.

### Relative Contraindications to High-Dose Cisplatin

Owing to the high prevalence of comorbid conditions in patients with head and neck cancer, many cases fall into this category (34). Here, more than in any of the two alternative clinical scenarios, physicians have to rely on local medical expertise including multidisciplinary tumor board meetings with an emphasis on patient engagement and shared decision making. Consequently, some practitioners opt for the standard of care, while others consider treatment plans recommended in the case of absolute contraindications to cisplatin (see below) [V, C]. Under such circumstances, lowering the peak concentration of cisplatin, as an important determinant for acute toxicity (nausea, vomiting, transaminase elevations, ototoxicity, serum creatinine increase), by either prolonging the infusion time (e.g., for 24 h) or reducing the single dose (e.g., weekly or daily administration or the 3 weekly schedule with a reduced dose) is justifiable as well (19, 35–38) [V, C]. If preference is given to weekly cisplatin, single doses of 40 mg/m^2^ are recommended to ensure that the majority of patients receive a cumulative dose of at least 200 mg/m^2^ (28). The latter proved difficult to be attained with lower single doses, and this could negatively impact on survival (27, 29). A split administration of 4 ×25 mg/m^2^ on 4 consecutive days instead of the standard 100 mg/m^2^ infusion is currently under investigation in the GORTEC 2015-02 trial.

### Absolute Contraindications to Cisplatin

This situation precludes both high-dose and low-dose cisplatin regimens. Combining carboplatin 70 mg/m^2^ and fluorouracil 600 mg/m^2^ daily for 4 days three times every 3 weeks, the Groupe d'Oncologie Radiothérapie Tête Et Cou (GORTEC) regimen was explored in two large randomized trials. Between 1994 and 1997, the GORTEC 94-01 trial randomly allocated 226 oropharyngeal cancer patients to receive either carboplatin/5-fluorouracil chemotherapy with conventionally fractionated radiotherapy or radiotherapy alone. The combined modality arm managed to significantly enhance overall survival and this benefit was maintained even after a median follow-up of 5.5 years (5 year rates: 22.4% vs. 15.8%) (39, 40). The GORTEC 99-02 recruited 840 patients between 2000 and 2007, distributing them evenly between conventional chemoradiotherapy with the same carboplatin/5-fluorouracil regimen as described above, accelerated radiotherapy with a slightly modified systemic treatment, and very accelerated radiotherapy alone. Compared with the latter approach, conventional chemoradiotherapy induced superior 3 year PFS (37.6% vs. 32.2%; HR 0.82, 95% CI 0.67–0.99, *p* = 0.041) and overall survival (42.6% vs. 36.5%; HR 0.81, 95% CI 0.67–0.99, *p* = 0.04), while the use of accelerated radiation did not provide any benefit in this trial. Importantly, giving all three cycles vs. less amount of chemotherapy seemed to generate better survival and distant control, and this could not be compensated by acceleration (41, 42). In both GORTEC 94-01 and GORTEC 99-02, the acute toxicity was the major downside of this type of conventional chemoradiotherapy. The rate of severe acute mucositis of about 70% was at the limit of clinical acceptance. In GORTEC 94-01, it almost doubled compared with the standard arm (71% vs. 39%). In summary, patients with a history of neurological, hearing, or renal comorbidities as the sole factors precluding cisplatin administration should be primarily considered for carboplatin/5-fluorouracil doublet [I, B].

Cetuximab is an immunoglobulin G1 chimeric monoclonal antibody against epidermal growth factor receptor (EGFR) and the only approved targeted agent in locoregionally advanced SCCHN. It is usually administered at an initial dose of 400 mg/m^2^ followed by weekly doses of 250 mg/m^2^. Serving as a possible alternative to platinum derivatives, the IMCL-9815 trial showed survival advantage with the addition of cetuximab to radiotherapy alone, primarily integrating altered fractionation and excluding oral cavity primaries (43). However, as suggested by several retrospective observations and recently confirmed by the De-ESCALaTE and RTOG 1016 trials, bioradiation with cetuximab should not be prioritized over the conventionally or altered fractionation cisplatin-based chemoradiation either in terms of efficacy or in terms of acute and late toxicity [II, B] (11, 12, 44–46). A similar conclusion has recently been suggested for the anti-tumor activity of the carboplatin/5-fluorouracil (vs. cetuximab) in patients who were not eligible for high-dose cisplatin, based on GORTEC 2007-01, showing superiority of this regimen plus cetuximab vs. cetuximab alone when combined with radiation (47). Moreover, since the publication of De-ESCALaTE and RTOG 1016, the recommendation for cetuximab as an adjunct to definitive radiotherapy has been weaker in patients with HPV-positive oropharyngeal cancer where it remains optional in the case of a contraindication for platinum-based chemotherapy [I, C]. In this situation, hyperfractionated radiotherapy alone might be a reasonable choice also (please see below).

Supported by limited scientific evidence, many practicing oncologist have been using single agent carboplatin as a less toxic substitute for cisplatin in these circumstances. Between 1988 and 1991, Jeremic et al. tested conventional radiotherapy (arm I) with or without daily administration of cisplatin (6 mg/m^2^, arm II) or carboplatin (25 mg/m^2^, arm III) (48). Fountzilas et al. utilized a similar three-arm design but with high-dose cisplatin (100 mg/m^2^) or carboplatin (area under the curve 7) administered every 3 weeks for a total of three infusions (49). In both studies, carboplatin had a significantly positive impact on overall survival with an acceptable toxicity profile most frequently in the form of bone marrow suppression. Nonetheless, the results should be interpreted with caution in view of the clearly insufficient number of patients treated with carboplatin in each of these trials (53 and 38, respectively) and its differing dose. Of note, according to the previously mentioned individual patient-based meta-analysis, only concomitant monochemotherapy with cisplatin or polychemotherapy including a platinum derivate or 5-fluorouracil gave a survival advantage when combined with radiotherapy, and this was not the case when carboplatin alone was used alone as a radiosensitizer [II, C] (17).

In selected cases where patient-related factors impede systemic treatment, altered fractionation radiotherapy alone should be pursued. The greatest survival gain can be achieved by hyperfractionation, especially in limited nodal disease (N0 and N1). This came forward in a large meta-analysis of 15 randomized trials comparing conventional radiotherapy with altered fractionation schedules in definitive treatment of non-metastatic SCCHN [I, B] (50). A recently published update corroborated its conclusions (51).

## Post-Operative Treatment Setting

### No or Only Relative Contraindications to High-Dose Cisplatin

With the exception of altered fractionation radiotherapy which should preferably not be delivered in the post-operative setting and the fact that data supporting a cumulative dose of 200 mg/m^2^ cisplatin in combination with conventional fractionation are extrapolated from the definitive setting [V, C], the remaining recommendations are equivalent to those pertinent to definitive treatment intent (52). Only one small randomized trial explored the outcome of adding weekly cisplatin to conventional radiotherapy in a sample of 88 participants. The statistically significant improvement of 5 year overall survival (13% vs. 36%), disease-free survival (23% vs. 45%), and locoregional control (55% vs. 70%) was accompanied by an increase in severe acute adverse events (16% vs. 41%). Of note, the used single (50 mg/m^2^) and cumulative (350–450 mg/m^2^) cisplatin doses exceeded those employed in current protocols, limiting thus the applicability of this weekly regimen in daily practice (53, 54). Data from other prospective and retrospective studies do not permit substituting the 3 weekly for a weekly schedule on a routine basis (23). For more on this subject, please refer to a separate chapter below.

### Absolute Contraindications to Cisplatin

In case the risk/benefit ratio strongly discourages from exposing patients to cisplatin, there is no adequate systemic replacement. In this context, patients with a high risk for recurrence should routinely receive conventional radiotherapy alone despite a paucity of randomized trials of post-operative radiotherapy vs. observation, originating from the fact that the concept of adjuvant therapy developed empirically [III, B] (55). Nevertheless, addressing the clinical need to potentiate treatment outcomes above all in patients in good clinical condition without other contraindications, several systemic agents have been recommended in combination with conventional radiotherapy in this setting. In the randomized RTOG 0234 phase II trial, 238 patients treated with post-operative radiotherapy were evenly divided into the following two arms, cetuximab with weekly cisplatin 30 mg/m^2^ or cetuximab with weekly docetaxel 15 mg/m^2^. With a median follow-up of 4.4 years, the latter regimen augmented overall survival relative to historical controls from the RTOG 9501 trial (2 year rates: 79% vs. 65%) with a 54% rate of severe acute mucositis [III, B] (56). Although some advantage has been suggested with the use of paclitaxel in definitive chemoradiation de-escalation trials in HPV-positive oropharyngeal cancer, this has not been tested in the post-operative setting (57, 58).

On the contrary, for single-agent cetuximab as an adjunct to post-operative radiotherapy no prospective evidence exists, and a recently published report on a small series of patients discouraged from its use here (59). Therefore, with the additional negative results of cetuximab/radiation in comparison with cisplatin/radiation in the definitive setting (see earlier) we do not recommend this approach [V, D]. Similarly, carboplatin/5-fluorouracil doublet has never been tested prospectively after curative resection. Nevertheless, it has been generally accepted as an adequate surrogate for high-dose cisplatin concurrent with definitive radiotherapy, and we assume comparable activity when extrapolated to the adjuvant setting [V, B]. However, for single-agent carboplatin, the rationale is weak at present. The only randomized trial in mucosal SCCHN was closed prematurely due to slow accrual and did not demonstrate any benefit with the addition of weekly carboplatin to adjuvant radiotherapy [II, D] (60). This is in line with another negative phase III trial performed in 321 patients with cutaneous SCCHN. The Trans-Tasman Radiation Oncology Group (TROG) 05.01 study provided high-quality data with a median follow-up of 60 months showing that potentiation by weekly low-dose carboplatin had no effect on survival or toxicity (61).

## Weekly vs. 3 Weekly Cisplatin

As alluded to above, 3 weekly high-dose cisplatin delivered concurrently with external beam radiotherapy remains the standard of care. This is in line with the results of a composite meta-analysis of 59 prospective trials enrolling altogether 5,582 patients (23, 24, 26). Although the weekly schedule produced less severe acute adverse events than three cycles of the standard regimen when combined with conventionally fractionated radiotherapy, no benefit could be observed in survival and late toxicity analyses. Of note, only two thirds of patients allocated to the high-dose arm could receive all three cycles (23). On the other hand, altered fractionation was associated with a significant advantage of two high-dose cisplatin cycles not only in terms of overall survival but in acute and late side effects. Here, the compliance with the standard regimen surpassed 90% (24). Moreover, in patients treated with adjuvant intent, two prospective trials comparing weekly vs. 3 weekly cisplatin are available. The first has been reported by Tsan et al. Among 55 randomly assigned patients followed for a median of 12 months, the 3 weekly regimen produced less acute toxicity, particularly severe mucositis, than weekly 40 mg/m^2^ cisplatin and proved also superiority in terms of reaching cumulative doses of at least 200 mg/m^2^ (62). Another proof against the routine use of weekly cisplatin was recently furnished by a single-center phase III trial from the Tata Memorial Cancer Centre in Mumbay, India, comparing weekly 30 mg/m^2^ vs. 3 weekly 100 mg/m^2^ cisplatin. Non-inferiority of the low-dose regimen could not be confirmed. The standard, high-dose group, showed significant gain in locoregional control, the primary objective (73.1% vs. 58.5% at 2 years, *p* = 0.014), albeit at the cost of an increased incidence of acute (84.6% vs. 71.6%, *p* = 0.006), but not late side effects (63).

In summary, the enhanced short-term tolerance of weekly cisplatin (i.e., less acute nausea, vomiting, transaminase elevations, ototoxicity, serum creatinine increase, and myelotoxicity) may be outweighed by compromised survival and a lack of improvement in late toxicity.

## Conclusions

With the advent of novel targeted drugs, particularly immunotherapy, the landscape of head and neck cancer management has been undergoing profound changes affecting the recurrent and/or metastatic setting in the first place. In locoregionally advanced disease, the limited efficacy and unfavorable safety profile of the standard cisplatin-based chemoradiation has prompted many attempts at improving or even substituting this regimen. Now, 15 years after the publication of the four seminal articles, there is finally some reason for optimism. The activity of the immune checkpoint inhibitors nivolumab and pembrolizumab has been demonstrated in at least three large phase III trials in recurrent/metastatic SCCHN and in 2019, the efficacy results of the first studies performed in the locoregionally advanced disease setting will be presented as well, including the PembroRad trial (NCT02707588) randomizing patients between definitive radiotherapy with pembrolizumab or cetuximab and the RTOG 3504 trial (NCT02764593) exploring different combinations of definitive radiotherapy, nivolumab, cisplatin, and cetuximab.

Recommendations presented in this review paper should not be understood as a dogmatic system of rules but rather a frame to guide clinical decision making in which we underscore an individual approach allowing for patient- and disease-related factors. The relevance of these instructions should pertain at least for some time even in the era of modern immunotherapy because the availability and accessibility of immunomodulating antibodies will unfortunately be restricted in many countries worldwide. In this situation, cisplatin will retain its significance and continue to represent a cost-effective and feasible modality saving patients' lives.

## Author Contributions

PS and JV drafted the manuscript. CS and JB contributed to writing of the manuscript. VC and RH contributed to the conception and reviewed the manuscript.

### Conflict of Interest Statement

PS: Consulting or advisory relationships: Merck KGaA, Servier. Honoraria received: Merck-Serono. VC: Consulting or advisory relationships: Merck-Serono, Eli Lilly, Servier, Celgene. Travel grants for congress: Bayer, Merck-Serono. JB: Advisory board: MSD, BMS, Merck, Astra-Zeneca. CS: Consulting and advisory services, speaking or writing engagements, public presentations: Pfizer, Merck. Direct research support: Roche. Non-financial interests: PI of EORTC 1420, non-remunerated member of the EORTC HNCG. JV: Has had in the last three years or has consulting/advisory relationships with: Amgen, AstraZeneca, Boehringer Ingelheim, Innate Pharma, Merck Serono, Merck Sharp & Dome Corp, PCI Biotech, Synthon Biopharmaceuticals, Debiopharm, and WntResearch and has received honoraria from Merck-Serono, Sanofi, and BMS. The remaining author declares that the research was conducted in the absence of any commercial or financial relationships that could be construed as a potential conflict of interest.
