# Supplementary material for: Cisplatin Eligibility Issues and Alternative Regimens in Locoregionally Advanced Head and Neck Cancer: Recommendations for Clinical Practice
Source: Front Oncol. 2019 Jun 11;9:464. doi: 10.3389/fonc.2019.00464 (PMC6579895; doi:10.3389/fonc.2019.00464)
Supplement: Supplementary file 1 [file Data_Sheet_1.pdf]

SUPPLEMENTARY APPENDIX

Supplementary Table S1

Supplementary Table S2

References cited in the Supplementary Tables S1 and S2

Contraindication to cisplatin	Study title or First author and year	Study type	Recommendation	Level of evidence and strength of recommendation
No	Adelstein, 2003 [1]	Ph III	3x 100 mg/m^2 every three weeks + conventional fractionation	[I, A]
	RTOG 91-11 [2]	Ph III		
	RTOG 0129 [3]	Ph III	2x 100 mg/m^2 every three weeks + altered fractionation	[I, B]
	Szturz, 2018 [4]	MA		
	RTOG 1016 [5]	Ph III		
	Sharma, 2010 [6]	Ph IIR	6-7x 40 mg/m^2 weekly + conventional fractionation	[II, C]
	Quon, 2011 [7]	Ph III		
	Ghosh-Laskar, 2016 [8]	Ph III		
	Szturz, 2017 [9]	MA		
	De-ESCALaTE HPV [10]	Ph III, SA	2x 100 mg/m^2 every three weeks + conventional fractionation	[IV, C]
	RTOG 0129 [3,11]	Ph III, SA		
	Otty, 2011 [12]	Rs		
	Spreafico, 2016 [13]	Rs		
Relative	Vermorken, 1982 [14]	Rs		[V, C]

	Vermorken, 1983 [15]	Rs	Lowering the peak cisplatin concentration	
	Vermorken, 1986 [16]	Rs		
	Kurihara, 1996 [17]	Rs		
Absolute	GORTEC 94-01 [18,19]	Ph III	Carboplatin/5-fluorouracil + conventional fractionation	[I, B]
	GORTEC 99-02 [20]	Ph III		
	IMCL-9815 [21]	Ph III	Cetuximab + conventional fractionation, except for HPV+ OPC	[II, B]
	GORTEC 2007-01 [22]	Ph III		
	De-ESCALaTE HPV [10]	Ph III	Cetuximab + conventional fractionation in HPV+ OPC	[I, C]
	RTOG 1016 [5]	Ph III		
	Jeremic, 1997 [23]	PsR	Carboplatin + conventional fractionation	[II, C]
	Fountzilas, 2004 [24]	Ph III		
	MACH-NC [25]	MA		
	MARCH [26,27]	MA	Hyperfractionated radiotherapy	[I, B]

Abbreviations: Ph, phase; MA, meta-analysis; Ph IIR, phase II randomized; SA, subgroup analysis; Rs, retrospective; PsR, prospective randomized; HPV+ OPC, human papillomavirus positive oropharyngeal cancer

Table S1. Overview of studies supporting the treatment recommendations in the definitive setting [1-27].

Contraindication to cisplatin	Study title or First author and year	Study type	Recommendation	Level of evidence and strength of recommendation
No	RTOG 95-01 [28]<br>EORTC 22931 [29]	Ph III<br>Ph III	3x 100 mg/m^2 every three weeks + conventional fractionation	[I, A]
	Bachaud 1991, 1996, [30,31]<br>Szturz, 2017 [9]	Ph III<br>MA	6-7x 40 mg/m^2 weekly + conventional fractionation	[II, C]
	De-ESCALaTE HPV [10]<br>RTOG 0129 [3,11]<br>Otty, 2011 [12]<br>Spreafico, 2016 [13]	Ph III, SA<br>Ph III, SA<br>Rs<br>Rs	2x 100 mg/m^2 every three weeks + conventional fractionation	[V, C]
Relative	Vermorken, 1982 [14]<br>Vermorken, 1983 [15]<br>Vermorken, 1986 [16]<br>Kurihara, 1996 [17]	Rs<br>Rs<br>Rs<br>Rs	Lowering the peak cisplatin concentration	[V, C]

Absolute	RTOG 0234 [32]	Ph III	Cetuximab/docetaxel<br>+ conventional fractionation	[III, B]
	GORTEC 94-01 [18,19]<br>GORTEC 99-02 [20]	Ph III<br>Ph III	Carboplatin/5-fluorouracil<br>+ conventional fractionation	[V, B]
	IMCL-9815 [21]	Ph III	Cetuximab<br>+ conventional fractionation	[V, D]
	Argiris, 2008 [33]	Ph III	Carboplatin<br>+ conventional fractionation	[II, D]
	Fletcher, 1970 [34]	Ps	Hyperfractionated radiotherapy	[III, B]

Abbreviations: Ph, phase; MA, meta-analysis; SA, subgroup analysis; Rs, retrospective; Ps, prospective

Table S2. Overview of studies supporting the treatment recommendations in the postoperative setting [3,9-21,28-34].

References cited in the Supplementary Tables S1 and S2

1. Adelstein DJ, Li Y, Adams GL, Wagner H Jr, Kish JA, Ensley JF, et al. An intergroup phase III comparison of standard radiation therapy and two schedules of concurrent chemoradiotherapy in patients with unresectable squamous cell head and neck cancer. J Clin Oncol. 2003; 21: 92-8.

2. Forastiere AA, Goepfert H, Maor M, Pajak TF, Weber R, Morrison W, et al. Concurrent chemotherapy and radiotherapy for organ preservation in advanced laryngeal cancer. N Engl J Med. 2003; 349: 2091-8.

3. Nguyen-Tan PF, Zhang Q, Ang KK, Weber RS, Rosenthal DI, Soulieres D, et al. Randomized phase III trial to test accelerated versus standard fractionation in combination with concurrent cisplatin for head and neck carcinomas in the Radiation Therapy Oncology Group 0129 trial: long-term report of efficacy and toxicity. J Clin Oncol. 2014; 32: 3858-66.

4. Szturz P, Wouters K, Kiyota N, Tahara M, Prabhash K, Noronha V, et al. Altered fractionation radiotherapy combined with concurrent low-dose or high-dose cisplatin in head and neck cancer: A systematic review of literature and meta-analysis. Oral Oncol. 2018; 76: 52-60. doi: 10.1016/j.oraloncology.2017.11.025.

5. Gillison ML, Trotti AM, Harris J, Eisbruch A, Harari PM, Adelstein DJ, et al. Radiotherapy plus cetuximab or cisplatin in human papillomavirus-positive oropharyngeal cancer (NRG Oncology RTOG 1016): a randomised, multicentre, non-inferiority trial. Lancet. 2019; 393: 40-50. doi: 10.1016/S0140-6736(18)32779-X.

6. Sharma A, Mohanti BK, Thakar A, Bahadur S, Bhasker S. Concomitant chemoradiation versus radical radiotherapy in advanced squamous cell carcinoma of oropharynx and nasopharynx using weekly cisplatin: a phase II randomized trial. Ann Oncol. 2010; 21: 2272-7. doi: 10.1093/annonc/mdq219.

7. Quon H, Leong T, Haselow R, Leipzig B, Cooper J, Forastiere A. Phase III study of radiation therapy with or without cis-platinum in patients with unresectable squamous or undifferentiated carcinoma of the head and neck: an intergroup trial of the Eastern Cooperative Oncology Group (E2382). Int J Radiat Oncol Biol Phys. 2011; 81: 719-25. doi: 10.1016/j.ijrobp.2010.06.038.

8. Ghosh-Laskar S, Kalyani N, Gupta T, Budrukkar A, Murthy V, Sengar M, et al. Conventional radiotherapy versus concurrent chemoradiotherapy versus accelerated radiotherapy in locoregionally advanced carcinoma of head and neck: Results of a prospective randomized trial. Head Neck. 2016; 38: 202-7. doi: 10.1002/hed.23865.

9. Szturz P, Wouters K, Kiyota N, Tahara M, Prabhash K, Noronha V, et al. Weekly Low-Dose Versus Three-Weekly High-Dose Cisplatin for Concurrent Chemoradiation in Locoregionally Advanced Non-Nasopharyngeal Head and Neck Cancer: A Systematic Review and Meta-Analysis of Aggregate Data. Oncologist. 2017; 22: 1056-1066. doi: 10.1634/theoncologist.2017-0015.

10. Mehanna H, Robinson M, Hartley A, Kong A, Foran B, Fulton-Lieuw T, et al. Radiotherapy plus cisplatin or cetuximab in low-risk human papillomavirus-positive oropharyngeal cancer (De-ESCALaTE HPV): an open-label randomised controlled phase 3 trial. Lancet. 2019; 393: 51-60. doi: 10.1016/S0140-6736(18)32752-1.

11. Ang K, Zhang Q, Wheeler RH, Rosenthal DI, Nguyen-Tan F, Kim H, et al. A phase III trial (RTOG 0129) of two radiation-cisplatin regimens for head and neck carcinomas (HNC): Impact of radiation and cisplatin intensity on outcome. J Clin Oncol. 2010; 28(suppl): Abstr 5507. doi: 10.1200/jco.2010.28.15_suppl.5507

12. Otty Z, Skinner MB, Dass J, Collins M, Mooi J, Thuraisingam K, Sabesan S. Efficacy and tolerability of weekly low-dose cisplatin concurrent with radiotherapy in head and neck cancer patients. Asia Pac J Clin Oncol. 2011; 7: 287-92. doi: 10.1111/j.1743-7563.2011.01405.x.

13. Spreafico A, Huang SH, Xu W, Granata R, Liu CS, Waldron JN, et al. Impact of cisplatin dose intensity on human papillomavirus-related and -unrelated locally advanced head and neck squamous cell carcinoma. Eur J Cancer. 2016; 67: 174-182. doi: 10.1016/j.ejca.2016.08.013.

14. Vermorken JB, van der Vijgh WJ, Klein I, Gall HE, Pinedo HM. Pharmacokinetics of free platinum species following rapid, 3-hr and 24-hr infusions of cis-diamminedichloroplatinum (II) and its therapeutic implications. Eur J Cancer Clin Oncol. 1982; 18:1069-74.

15. Vermorken JB, Kapteijn TS, Hart AA, Pinedo HM. Ototoxicity of cis-diamminedichloroplatinum (II): influence of dose, schedule and mode of administration. Eur J Cancer Clin Oncol. 1983; 19:53-8.

16. Vermorken JB, van der Vijgh WJ, Klein I, Gall HE, van Groeningen CJ, Hart GA, Pinedo HM. Pharmacokinetics of free and total platinum species after rapid and prolonged infusions of cisplatin. Clin Pharmacol Ther. 1986; 39:136-44.

17. Kurihara N, Kubota T, Hoshiya Y, Otani Y, Ando N, Kumai K, Kitajima M. Pharmacokinetics of cis-diamminedichloroplatinum (II) given as low-dose and high-dose infusions. J Surg Oncol. 1996; 62: 135-8.

18. Calais G, Alfonsi M, Bardet E, Sire C, Germain T, Bergerot P, et al. Randomized trial of radiation therapy versus concomitant chemotherapy and radiation therapy for advanced-stage oropharynx carcinoma. J Natl Cancer Inst. 1999; 91: 2081-6.

19. Denis F, Garaud P, Bardet E, Alfonsi M, Sire C, Germain T, et al. Final results of the 94-01 French Head and Neck Oncology and Radiotherapy Group randomized trial comparing radiotherapy alone with concomitant radiochemotherapy in advanced-stage oropharynx carcinoma. J Clin Oncol. 2004; 22: 69-76.

20. Bourhis J, Sire C, Graff P, Grégoire V, Maingon P, Calais G, et al. Concomitant chemoradiotherapy versus acceleration of radiotherapy with or without concomitant chemotherapy in locally advanced head and neck carcinoma (GORTEC 99-02): an open-label phase 3 randomised trial. Lancet Oncol. 2012; 13: 145-53. doi: 10.1016/S1470-2045(11)70346-1.

21. Bonner JA, Harari PM, Giralt J, Azarnia N, Shin DM, Cohen RB, et al. Radiotherapy plus cetuximab for squamous-cell carcinoma of the head and neck. N Engl J Med. 2006; 354: 567-78.

22. Tao Y, Auperin A, Sire C, Martin L, Khoury C, Maingon P, et al. Improved Outcome by Adding Concurrent Chemotherapy to Cetuximab and Radiotherapy for Locally Advanced Head and Neck Carcinomas: Results of the GORTEC 2007-01 Phase III Randomized Trial. J Clin Oncol. 2018: JCO2017762518. doi: 10.1200/JCO.2017.76.2518.

23. Jeremic B, Shibamoto Y, Stanisavljevic B, Milojevic L, Milicic B, Nikolic N. Radiation therapy alone or with concurrent low-dose daily either cisplatin or carboplatin in locally advanced unresectable squamous cell carcinoma of the head and neck: a prospective randomized trial. Radiother Oncol. 1997; 43: 29-37.

24. Fountzilas G, Ciuleanu E, Dafni U, Plataniotis G, Kalogera-Fountzila A, Samantas E, et al. Concomitant radiochemotherapy vs radiotherapy alone in patients with head and neck cancer: a Hellenic Cooperative Oncology Group Phase III Study. Med Oncol. 2004; 21: 95-107.

25. Pignon JP, le Maître A, Maillard E, Bourhis J; MACH-NC Collaborative Group. Meta-analysis of chemotherapy in head and neck cancer (MACH-NC): an update on 93 randomised trials and 17,346 patients. Radiother Oncol. 2009; 92: 4-14. doi: 10.1016/j.radonc.2009.04.014.

26. Bourhis J, Overgaard J, Audry H, Ang KK, Saunders M, Bernier J, et al. Hyperfractionated or accelerated radiotherapy in head and neck cancer: a meta-analysis. Lancet. 2006; 368: 843-54.

27. Lacas B, Bourhis J, Overgaard J, Zhang Q, Grégoire V, Nankivell M, et al. Role of radiotherapy fractionation in head and neck cancers (MARCH): an updated meta-analysis. Lancet Oncol. 2017; 18: 1221-37. doi: 10.1016/S1470-2045(17)30458-8.

28. Cooper JS, Pajak TF, Forastiere AA, Jacobs J, Campbell BH, Saxman SB, et al. Postoperative concurrent radiotherapy and chemotherapy for high-risk squamous-cell carcinoma of the head and neck. N Engl J Med. 2004; 350: 1937-44.

29. Bernier J, Domenge C, Ozsahin M, Matuszewska K, Lefèbvre JL, Greiner RH, et al. Postoperative irradiation with or without concomitant chemotherapy for locally advanced head and neck cancer. N Engl J Med. 2004; 350: 1945-52.

30. Bachaud JM, David JM, Boussin G, Daly N. Combined postoperative radiotherapy and weekly cisplatin infusion for locally advanced squamous cell carcinoma of the head and neck: preliminary report of a randomized trial. Int J Radiat Oncol Biol Phys. 1991; 20: 243-6.

31. Bachaud JM, Cohen-Jonathan E, Alzieu C, David JM, Serrano E, Daly-Schveitzer N. Combined postoperative radiotherapy and weekly cisplatin infusion for locally advanced head and neck carcinoma: final report of a randomized trial. Int J Radiat Oncol Biol Phys. 1996; 36:999-1004.

32. Harari PM, Harris J, Kies MS, Myers JN, Jordan RC, Gillison ML, et al. Postoperative chemoradiotherapy and cetuximab for high-risk squamous cell carcinoma of the head and neck: Radiation Therapy Oncology Group RTOG-0234. J Clin Oncol. 2014; 32: 2486-95. doi: 10.1200/JCO.2013.53.9163.

33. Argiris A, Karamouzis MV, Johnson JT, Heron DE, Myers E, Eibling D, et al. Long-term results of a phase III randomized trial of postoperative radiotherapy with or without carboplatin in patients with high-risk head and neck cancer. Laryngoscope 2008; 118: 444-9.

34. Fletcher GH, Evers WT. Radiotherapeutic management of surgical recurrences and postoperative residuals in tumors of the head and neck. Radiology. 1970; 95: 185-8.
